# Rehabilitation effect of omega-3 polyunsaturated fatty acids on exercise-induced muscle damage: a mini-review

**DOI:** 10.3389/fnut.2026.1932144

**Published:** 2026-09-08

**Authors:** Jianan Ni, Lulu Shen, Song Gao

**Affiliations:** 1 College of Physical Education and Health Sciences, Zhejiang Normal University, Jinhua, China; 2 Jinhua Deren Rehabilitation Equipment Corporation, Jinhua, China

**Keywords:** anti-inflammatory, exercise-induced muscle damage, long-chain polyunsaturated acids, myosatellite cells, plasma membrane repair

## Abstract

Omega-3 polyunsaturated fatty acids (PUFAs) are vital components of life activities, and the human body is incapable of synthesizing them; therefore, they must be obtained from the diet. Recent research indicates that these substances can influence not only the metabolic reaction to exercise and skeletal muscle function, but also the functional performance of exercise training. A growing body of research has indicated that it can facilitate the recovery process of exercise-induced muscle damage (EIMD); however, the underlying mechanisms remain to be fully elucidated. In addition, the potential anti-inflammatory and antioxidant activities of omega-3 polyunsaturated fatty acids have the capacity to engender health benefits and performance enhancement, particularly among individuals who partake in physical activities. This paper summarizes the effect of omega-3 PUFAs on EIMD and analyses the potential mechanism of omega-3 PUFAs in EIMD repair.

## Introduction

Exercise-induced muscle damage (EIMD) is the term given to the injury of muscle fibers, tendons or surrounding tissues caused by excessive exercise, external impact or improper posture, including strain, contusion and tear (1). The principal findings of EIMD consist of the destruction of myofibrils, a secondary inflammatory reaction, and oxidative stress. The manifestation of EIMD is contingent on the nature of the exercise, encompassing endurance, resistance, and high-intensity interval training modalities. The intensity and duration of the exercise also serve as pivotal factors in the expression of this condition. Commonly associated with EIMD are manifestations such as diminished muscle function, protracted muscle soreness, and diminished joint activity (2, 3). It is evident that EIMD will have a detrimental effect on the performance of training and competitive events (4). Nevertheless, there is an absence of effective methodologies for the amelioration of symptoms associated with EIMD. The nutrition supplement strategy has gradually attracted researchers' attention (3). The acute recovery period of 96 h following exercise is widely considered to be pivotal in optimizing sports performance, with nutritional strategies having the capacity to promote the recovery process of EIMD (5).

It is a widely acknowledged fact that high-intensity centrifugal exercise is associated with muscle damage (6). Research has demonstrated that omega-3 PUFAs have the capacity to enhance the structural stability of muscle cell membranes (7). Inflammation represents a pivotal process in the repair and regeneration of muscle tissue, and eicosapentaenoic acid (EPA) and docosahexaenoic acid (DHA) have been demonstrated to accelerate the recovery process by modulating immune function. The influence of these cells on immune regulation is characterized by an increase in interleukin-2 levels (8, 9). Research has demonstrated that when amateur male athletes consume a specific dose of fish oil prior to performing eccentric exercise, it results in a reduction of post-exercise concentrations of tumor necrosis factor-α and prostaglandin E2 (10). An experiment involving 45 male amateurs demonstrated that the consumption of 1.8 grams of fish oil prior to each eccentric exercise session led to a significant reduction in the concentrations of tumor necrosis factor-α and prostaglandin E2 immediately after exercise, as well as at 24 and 48 h post-exercise. Furthermore, a substantial decline in the concentrations of interleukin-6, CK, and myoglobin was observed at 24 and 48 h post-exercise (10).

Omega-3 PUFAs represent a significant class of functional high-value fatty acids, encompassing α-linolenic acid (ALA), EPA and DHA (11). The primary dietary sources of this substance are vegetable oil and marine fatty fish. In recent years, there has been a significant advancement in the production of omega-3 PUFAs by microalgae, filamentous fungi, oil-producing yeast and other microorganisms. This development has addressed the issues of supply shortage and unstable quality, thereby expanding the application scope of omega-3 PUFAs. Omega-3 PUFAs have been demonstrated to have significant effects on the prevention of cardiovascular diseases, neurodegenerative diseases, depression, cancer, promotion of infant nerve development, wound healing and tissue repair (12–14). The molecular mechanism of action of omega-3 PUFAs involves the alleviation of nervous and systemic inflammation, the maintenance of cell structure and function, and the activation of immune signals (15, 16). Omega-3 PUFAs have garnered significant attention in the field of sports nutrition due to their multifaceted benefits, which include anti-inflammatory and antioxidant properties, in addition to their capacity to stimulate muscle synthesis. These properties have led to the frequent utilization of omega-3 PUFAs as a component of sports nutritional supplements, with numerous studies highlighting their potential in accelerating the recovery process of EIMD (17, 18). Research has demonstrated that the supplementation of omega-3 PUFAs, specifically EPA and DHA, is advantageous in accelerating the recovery process of EIMD (19–21). This study summarized the role and potential mechanisms of omega-3 PUFAs in promoting the recovery of EIMD. The mechanisms encompass the myogenic repair mechanism of satellite cells in the skeletal muscle cell membrane, the repair mechanism of the skeletal muscle plasma membrane, and the anti-inflammatory and antioxidant repair mechanisms closely related to organelles. A comprehensive understanding of omega-3 PUFAs is conducive to the development of novel nutritional supplement strategies.

## Omega-3 polyunsaturated fatty acids and EIMD repair

Omega-3 PUFAs are defined as long-chain polyunsaturated acids, encompassing ALA, stearic acid, EPA, docosapentaenoic acid (DPA) and DHA. The human body is incapable of synthesizing omega-3 fatty acids, which must therefore be obtained from food sources. The conversion of ALA to EPA and DHA by the human body is a complex process, with a ratio of only 8%−12% of ALA being converted into EPA, and a lower ratio of less than 1% being converted into DHA (22). This emphasizes the importance of dietary supplementation with EPA and DHA. Marine life constitutes the primary source of omega-3 PUFAs (EPA and DHA) supplements, encompassing fish oil (predominantly in the form of ethyl ester or acyl glycerol), krill oil (in concentrated form of triacylglycerol and phospholipids), and novel renewable algae, fungi, and microbial oils (16). Plant seeds have been demonstrated to be a significant source of omega-3 PUFAs (ALA) supplements, with linseed oil, chia seed oil and rapeseed oil being notable examples. These seeds function as precursors in the human body's synthesis of long-chain PUFAs (23). A series of experimental studies have confirmed that omega-3 PUFAs can promote the recovery of EIMD, including muscle function, slight injury of skeletal muscle, oxidative stress and inflammatory reaction. Research findings concerning the relationship between Omega-3 polyunsaturated fatty acids and EIMD repair are presented in Table 1.

**Table 1:** Effects of omega-3 PUFAs supplementation with a focus on EIMD repair-related outcomes in athletes and amateurs.

References	Population (*n*)	Duration (weeks)	Dose EPA/DHA (g/d)	Exercise intervention/test	Findings
	Sex age ±SD (years)				
Tsuchiya et al. (21)	Healthy (24) M 19.5 ± 0.8	8 weeks	0.6 g EPA 0.26 g DHA	Eccentric contraction-induced muscle damage Changes in the MVC torque ROM Upper arm circumference, muscle soreness CK, myoglobin, IL-6, and TNF-α levels	MVC 2–5 days after exercise↑ ROM at 1–5 days after exercise↑ Muscle soreness 3 days after exercise ↓ Increase in serum IL-6 levels↓
Jakeman et al. (27)	High-intensity intermittent training athletes (27) M 26 ± 4	Acute	Group 1: 0.75 g EPA, 0.05 g DHA per 10 kg body weight Group 2: 0.15 g EPA, 0.1 g DHA per 10 kg body weight	Recovery strategy following 100 plyometric drop jumps	Squat jump performance, increased in group1 CMJ performance, unchanged Functional and perceptual indices, unchanged Perceived soreness, unchanged
Jouris et al. (24)	Healthy (11) M and F 18–60	1 week	2 g EPA 1 g DHA	Eccentric strength exercise Inflammation Soreness ratings Arm circumference and volume Temperature	Soreness↓ Arm circumference, no difference Arm volume, no difference Skin temperature, no difference
Tinsley et al. (28)	Healthy (17) F 22.5 ± 1.8 and 24.7 ± 3.6	1 week	6 g FO (5:1 EPA:DHA)	Post resistance exercise muscle soreness Soreness during functional movements and limb circumferences	Muscle soreness, no difference
Da Boit et al. (9)	Healthy (37) M and F 25.8 ± 5.3	6 weeks	0.24 g EPA 0.12 g DHA	Maximal incremental exercise test and cycling time trial. Post-exercise immune function and performance Plasma IL-6 and TBARS concentrations and, erythrocyte fatty acid composition NK cell cytotoxic activity and PBMC IL-2, IL-4, IL-10, IL-17 IFNγ production	PBMC IL-2 and NK cell cytotoxic activity 3 h post-exercise↑ Plasma IL-6 and TBARS, PBMC IL-4, IL-10, IL-17 and IFNγ production, along with performance and physiological measures during exercise, no difference
Kyriakidou et al. (20)	Healthy (14) M 25.07 ± 4.05	4 weeks	3 g/day omega-3 PUFAs	downhill running protocol (60 min, 65% VO_2max_, – 10% gradient) CK, IL-6 and TNF-α, perceived muscle soreness, MVIC and peak power	Muscle soreness ↓
Corder et al. (29)	Healthy (27) F 20–60	1 week	3 g/day DHA	Eccentric exercise: subjects performed four sets of eccentric biceps curls on a preacher bench with the non-dominant arm Markers of inflammation and soreness	Palpation soreness rating ↓ Inability to fully extend elbow at 48 h follow-up ↓ Passive elbow joint angle, arm circumference, skin temperature, C-reactive protein, no difference

M, male; F, female; MVC, maximum voluntary contraction; ROM, range of motion; CK, creatine kinase; IL-6, interleukin-6; TNF-α, tumor necrosis factor-α; FO, fish oil; MVIC, maximal voluntary isometric contraction; NK, natural killer; PBMC, peripheral blood mononuclear cell; TBARS, thiobarbituric acid reactive substances. ↑, increased; ↓, decreased.

## Fish oil containing omega-3 PUFAs can promote the recovery of EIMD

Recent studies have confirmed that fish oil, which is rich in omega-3 PUFAs, can promote the recovery of EIMD. Tsuchiya et al. (21) conducted a double-blind experiment on 22 men and confirmed that continuous supplementation of omega-3 PUFAs for a period of 4 weeks can accelerate the recovery of muscle injury caused by high-intensity centrifugal contraction. Four weeks of fish oil supplementation (600 mg/day of EPA and 260 mg/day of DHA) has been shown to increase the serum EPA level of adult men without training experience, and to reduce the decrease in range of motion (ROM) of the elbow flexors immediately after eccentric exercise. Furthermore, the serum creatine kinase level was also found to decrease 48 h after exercise (21). In a control experiment by Jouris et al. (24), the control group was subjected to a 14-week dietary restriction on omega-3 PUFAs, while the experimental group consumed 3,000 mg/day of omega-3 PUFAs for seven consecutive days. This was followed by the performance of two maximum eccentric biceps brachii bends. Following a 48-h period, the muscle pain levels and swelling degrees of the two groups were evaluated. The present study found that supplementation of 3,000 mg/d Omega-3 PUFAs could minimize severe and delayed muscle soreness caused by strenuous eccentric exercise. The present study investigates the effects of 4 weeks of fish oil supplementation (EPA 2145 mg/d, DHA 858 mg/d) on biomarkers in healthy adult males following a single bout of downhill running (60 min, 65% VO_2_max). The results demonstrate a reduction in serum levels of interleukin-6 (IL-6) and creatine kinase and delayed onset muscle soreness 24 h after exercise. Additionally, the supplementation increased the maximum voluntary contraction (MVC) (20). It has been demonstrated that other, more protracted fish oil supplementation cycles have the capacity to enhance the muscle function (comprising muscle strength, ROM, stiffness, etc.) of skeletal muscle following high-intensity exercise, whilst concomitantly attenuating the levels of skeletal muscle micro-injury, oxidative stress and inflammation (19, 25, 26).

## Acute and short-term fish oil supplementation cycles are also considered to promote the recovery process of EIMD

Jakeman et al. (27) found that adult males immediately ingested 1 g of fish oil per 10 kg of body mass following 100 squats. It has been established that 1 g of fish oil contains 750 mg of EPA and 50 mg of DHA. This can improve the performance of squats and reverse jumps after exercise. As demonstrated by Jouris et al. (24), a 7-day course of fish oil supplementation (1,000 mg/day) has been shown to reduce muscle soreness in healthy men and women following eccentric exercise of the elbow flexors for a period of 48 h. Similarly, a study by Tinsley et al. (28) found that 7 days of fish oil supplementation (6 g/day) reduced the static and functional muscle soreness of adult women after resistance exercise of the elbows and lower limbs for a period of 48 h. In addition, Corder et al. (29) found that 9 days of DHA supplementation (3,000 mg/day) reduced the degree of muscle soreness and stiffness 48 h after eccentric exercise of the elbow flexors in young women, and inhibited the reduction of ROM.

Krill oil supplement containing ω-3 PUFAs can also promote the recovery of EIMD. A study by Skarpańska-Stejnborn et al. (30) found that 6-week krill oil (1 g/d) supplementation can reduce the level of thiobarbituric acid reactive substances in rowers following intensive exercise for 24 h. Another study found that 6-week krill oil (2 g/d) supplementation can improve natural killer cell activity in young athletes following exhaustive exercise and reduce the production of interleukin-2 (IL-2) in peripheral blood mononuclear cells after three hours of exercise (9).

## Effect of omega-3PUFA supplementation on muscle mass and volume

As demonstrated by both *in vivo* (31) and *in vitro* (32) studies, the muscle protein synthesis of young and old subjects increased significantly after administration of 4 g omega-3 PUFAs on a daily basis for a period of 8 weeks (33). In a similar manner, 6-month supplementation (3.36 g/day) resulted in an augmentation of muscle mass (+3.6%) and strength (+4%) in elderly subjects (34). A number of meta-analyses have indicated that omega-3PUFA supplementation exerts a beneficial effect in terms of maintaining muscle mass. However, the certainty of these results is limited, and the optimal dosage remains to be established. A recent meta-analysis found that, in comparison with the control group, the skeletal muscle mass of elderly individuals aged 60 and above in the omega-3PUFA group increased marginally by 0.33 kg (35). The estimated value is derived from a synthesis of five small studies exhibiting minimal heterogeneity (omega-3 PUFA group, *n* = 103; control group, *n* = 99). Specifically, enhancement was only observed in subjects who received a daily intake of more than 2 g of omega-3 PUFA, with the combined effect resulting in a 0.67 kg change, thereby indicating a dose-effect relationship. However, it is important to exercise caution when interpreting these findings, as one of the included studies utilized α-linolenic acid supplements in place of omega-3 PUFA (36). In addition, the meta-analysis encompassed a dietary intervention focused on the consumption of omega-3 PUFA, which revealed the proportion of omega-6 to omega-3 PUFA (37, 38). Consequently, the precise impact of omega-3 PUFA as a supplement remains ambiguous. Nevertheless, the positive effect of omega-3PUFA was confirmed in another meta-analysis, which compiled eight randomized controlled trials with 406 elderly participants (39). However, these subjects primarily exhibit significant comorbidities, including cancer, chronic obstructive pulmonary disease and morbid obesity undergoing surgery (40–42). It is evident that these patients already exhibit chronic inflammation, a factor which may elucidate the amplified response to omega-3 PUFA (43). Furthermore, these studies exhibit substantial variations in the forms of omega-3PUFA supplements, ranging from fish oil to oral multi-nutrient supplements. The duration of the program is subject to variation, ranging from a minimum of 12 days to a maximum of 4 months. However, no sensitivity analysis has been conducted to ascertain the impact of these methodological discrepancies.

A number of other studies have reported favorable outcomes with regard to the positive performance of muscle recovery and training adaptation (29, 44, 45). The ingestion of omega-3 PUFAs has been demonstrated to have a beneficial effect on the reduction of muscle strength loss and reduced range of motion, as well as on blood markers of inflammation (TNF-a) and markers of muscle injury (myoglobin, creatine kinase and skeletal muscle troponin I) (25). Consequently, omega-3 PUFAs have the potential to mitigate anabolic resistance and may be utilized as a therapeutic agent in the management of catabolic processes.

## Mechanism of omega-3 polyunsaturated fatty acids promoting EIMD repair

### Omega-3 PUFA and muscle satellite cells

In the event of a muscle injury, the activation of complex reactions within the skeletal muscle is initiated, thereby facilitating the process of tissue repair (46). The muscle satellite cell is a unique cell type found in skeletal muscle tissue. It is regarded as a specific stem cell of skeletal muscle and possesses pluripotency, which is the capacity to differentiate into various cell types. Satellite cells of the muscle have the capacity to differentiate into a wide variety of cell types, including muscle cells. These cells have the ability to fuse with damaged skeletal muscle fibers, thereby playing a pivotal role in the process of skeletal muscle self-repair (47). The proliferation and differentiation of muscle satellite cells can be regulated by nutrients (48). Omega-3 PUFAs have been demonstrated to possess the capacity to modulate gene expression, mitochondrial biogenesis and inflammation. Consequently, they are regarded as playing a regulatory role in the myogenic regeneration of skeletal muscle and the promotion of skeletal muscle repair (49).

It has been demonstrated that EPA and DHA play a pivotal role in the process of myogenic activation of muscle satellite cells (48). Pax7 expression is the characteristic of muscle satellite cells at rest. However, C2C12 myoblasts cultured with EPA and/or DHA for 24 or 48 h have been observed to exhibit a decrease in Pax7 expression and an increase in myostatin expression. This suggests that EPA and DHA induce the transition of muscle satellite cells from a state of rest to activation (50).

Omega-3 PUFAs have been demonstrated to promote myogenic differentiation by regulating mitochondrial biogenesis. Following skeletal muscle injury, the oxygen consumption rate and glycolysis rate of muscle satellite cells will increase (51). Treatment of myotubes with 50 μmol/L EPA+DHA for 24 h resulted in increased expression of PGC-1α, NRF1, and TFAM mRNA, and an increase in the ratio of mtDNA to nuclear DNA. These findings suggest that EPA + DHA can enhance mitochondrial biogenesis during myogenesis (52). Løvsletten et al. (53) isolated muscle satellite cells from healthy human muscles and induced them to differentiate into multinucleated myotubes. Following a 24-h treatment period with 100 μmol/L EPA, an increase in both the mitochondrial oxygen consumption rate and proton leakage was also observed. This finding indicates that EPA enhances the function of mitochondria during myogenesis.

### Omega-3 PUFAs and skeletal muscle plasma membrane

The skeletal muscle plasma membrane constitutes a physical barrier between muscle cells and the internal environment, with its primary function being to maintain muscle cell homeostasis. It has been demonstrated that mechanical stimulation, induced by strenuous physical exertion, in conjunction with oxidative stress and an inflammatory reaction that ensues post-exercise, can result in the disruption of the structural integrity of the skeletal muscle plasma membrane. This phenomenon is accompanied by a concomitant reduction in the membrane's capacity for autonomous repair mechanisms. Damage to the skeletal muscle plasma membrane may result in the exudation of CK from skeletal muscle cells, which can ultimately lead to cell death. This process is recognized as the primary cause of EIMD.

The supplementation of omega-3 PUFAs has been demonstrated to have a beneficial effect on the plasma membrane damage of skeletal muscle caused by high-intensity exercise, with the potential to promote the repair of said membranes.

DHA and EPA are components of significant importance to the plasma membranes of skeletal muscle, and dietary omega-3 PUFAs (EPA and DHA) are considered to be able to maintain their integrity by doping into skeletal muscle plasma membrane (54). Supplementation with omega-3 PUFAs has been demonstrated to permeate the plasma membrane of skeletal muscle, thereby maintaining its structural integrity and optimal fluidity (54). In a similar manner, lecithin and sphingomyelin, which are vital components of the plasma membrane, possess a high number of unsaturated double bonds in their internal structures, which readily react with oxygen free radicals. The ingestion of DHA and EPA has been demonstrated to enhance the double bond structure within the membrane of muscle cells, thereby augmenting the antioxidant capacity (55).

In contrast, it was determined that supplementation with omega-3 PUFAs, derived from krill oil, can enhance the unsaturated fatty acid composition of skeletal muscle plasma membranes. This finding suggests a potential mechanism by which omega-3 PUFAs may promote membrane fluidity and facilitate the recovery process following exhaustive exercise in murine models (54). It is important to note that the research of McGlory et al. (56) showed that the incorporation of omega-3 PUFAs into skeletal muscle cells requires a minimum of 2 weeks, and that the absorption of omega-3 PUFAs by skeletal muscle is maximized after a period of more than 4 weeks. However, the present study detected the content of omega-3PUFAs in skeletal muscle, rather than in the cell membrane of said muscle tissue. Furthermore, studies have demonstrated that a high level of omega-3 PUFAs in blood can prevent the decrease of maximum voluntary contraction (MVC) and joint flexibility after eccentric exercise in young men (57). Consequently, the supplementation of short-term/acute omega-3 PUFAs may exert effects not only on skeletal muscle, but also on the body as a whole.

### Omega-3 PUFAs and anti-inflammatory ability

The second stage of EIMD is characterized by an inflammatory response and oxidative stress following exercise, resulting in temporary loss of muscle function and increased muscle soreness. As the second stage is conducive to optimal bodily adaptation (comprising muscle remodeling and enhanced skeletal muscle performance), it is also designated the recovery stage (58). However, it has been demonstrated that excessive immunosuppression and oxidative stress after exercise can lead to damage to cellular proteins, lipids and DNA, thereby hindering the recovery of skeletal muscle (59). The moderate improvement of the body's anti-inflammatory and antioxidant capabilities after exercise is an important factor in promoting the recovery of EIMD (8).

The administration of omega-3 PUFAs has been demonstrated to enhance the body's anti-inflammatory responses following exercise, thereby facilitating the recovery process of EIMD. A study by Gray et al. (8) found that the ingestion of 3 g of deep-sea fish oil (EPA and DHA) on a daily basis for a period of 6 weeks can significantly improve the activity of PBMC IL-2 (peripheral blood monocyte) and NK (macrophage) cells in men following exercise. As demonstrated in research, omega-3 polyunsaturated fatty acids have been found to be associated with cytokines and other proteins involved in the process of inflammation (60). Research has demonstrated that Omega-3 polyunsaturated fatty acids (mainly EPA and DHA) have the capacity to inhibit NF-κB signaling pathway and reduce the production of inflammatory factors by decreasing the phosphorylation level of IκBα (61). In the context of immune responses, two primary subgroups of Th cells, designated Th1 cells and Th2 cells, have been identified as secreting distinct types of cytokines. Th1 cells primarily secrete pro-inflammatory factors, including IFN-γ, IL-1β, TNF-α, IL-6, and others. Th2 cells primarily secrete anti-inflammatory factors, including IL-4, IL-5, IL-10, and others. Omega-3 polyunsaturated fatty acids have been demonstrated to influence the equilibrium of Th1 and Th2 cells by modulating the anti-inflammatory and immunosuppressive cytokine network in the post-exercise context. This results in a reduction of the Th1/Th2 ratio within the body, thereby enhancing the anti-inflammatory response (62, 63).

In experimental studies conducted on animals, the chronic supplementation of omega-3 PUFAs has been demonstrated to exert a positive influence on neuromuscular activity. This phenomenon may be attributable to the fact that DHA constitutes the fundamental component of the phospholipid in neuronal membranes and serves as the foundation for neuronal pathways. The precise mechanism by which omega-3 PUFAs influence protein synthesis in muscle is not yet fully elucidated. However, it has been demonstrated that they induce partial mediation by increasing the activation of the mTOR-ribosomal protein S6 kinase β-1 signaling pathway (64). This pathway is regarded as a vital regulatory point for muscle cell growth. This results in the stimulation of anabolism and the induction of an increase in the rate of muscle protein synthesis (65). Consequently, omega-3 PUFAs have the potential to impede the catabolic process, thus rendering them a promising therapeutic agent for the treatment of sarcopenia (65).

## Conclusion

EIMD represents the most prevalent form of such injury, typically manifesting subsequent to high-intensity or protracted exercise regimens. This study summarized the effects of omega-3 PUFAs on EIMD and examined the potential mechanisms by which omega-3 PUFAs may promote EIMD repair, including the activation of myogenic repair of muscle satellite cells, the enhancement of plasma membrane repair of skeletal muscle, and the augmentation of anti-inflammatory and antioxidant effects. The promotion of myogenic regeneration and self-repair of skeletal muscle is achieved by regulating gene expression and mitochondrial biogenesis. The physical properties of skeletal muscle cells, such as fluidity, are regulated by changing the phospholipid composition of cell membranes, thus promoting the repair of skeletal muscle plasma membrane injury. The influence of the polarization state of macrophages can promote the recovery of immune suppression of EIMD.

Nevertheless, there are still many problems in related research at present, which require further study and discussion. It is evident that there are discernible discrepancies in the dosage and duration of omega-3 PUFAs supplements employed in the various studies. Furthermore, the duration of the return of the concentration of omega-3 PUFAs in skeletal muscle and blood to the baseline level following the cessation of supplementation remains ambiguous. The optimal dosage of omega-3 PUFAs for the alleviation of inflammation, the promotion of the recovery of EIMD, and the maximization of the incorporation of DHA and EPA into skeletal muscle cells and skeletal muscle cell membranes remains to be ascertained. Consequently, the optimal supplementary scheme cannot be determined. The ratio of EPA to DHA in omega-3 PUFAs supplements is not uniform, and the independent roles of EPA and DHA should also be considered. Furthermore, the dosage, duration and types of different exercise modes should be considered. For instance, in the context of long-term endurance sports such as marathon running and rowing, there is robust evidence for the occurrence of oxidative stress and inflammatory reactions in the hours following exercise. This finding underscores the importance of a prolonged supplementation period, complemented by the incorporation of omega-3 PUFAs, which have been demonstrated to possess enhanced antioxidant properties. Athletes participating in explosive events must ensure that their muscles remain functional following periods of physical exertion. In such cases, the administration of short-term, high-dose omega-3 PUFAs supplementation is a more appropriate course of action.
